# Successful treatment of restless leg syndrome with the traditional herbal medicines Dangguijakyak-san and Shihogyeji-tang: A case report (CARE-compliant)

**DOI:** 10.1097/MD.0000000000026800

**Published:** 2021-08-06

**Authors:** Yuna Seo, Chul Jin, Bo-Hyoung Jang, Jin Pyeong Jeon, Ye-Seul Lee, Seung-Bo Yang, Woo-Sang Jung, Sang-Kwan Moon, Ki-Ho Cho, Seungwon Kwon

**Affiliations:** a Department of Cardiology and Neurology, College of Korean Medicine, Kyung Hee University, Seoul, Republic of Korea; b Department of Preventive Medicine, College of Korean Medicine, Kyung Hee University, Seoul, Republic of Korea; c Department of Neurosurgery, Chuncheon Sacred Heart Hospital, Hallym University Medical Center, Hallym University College of Medicine, Chuncheon, Republic of Korea; d Jaseng Spine and Joint Research Institute, Jaseng Medical Foundation, Seoul, Republic of Korea; e Department of Korean Internal Medicine, College of Korean Medicine, Gachon University, Seongnam, Republic of Korea.

**Keywords:** Dangguijakyak-san, restless leg syndrome, Shihogyeji-tang, traditional East Asian herbal medicine, Willis-Ekbom disease

## Abstract

**Rationale:**

Dopamine replacement is currently the standard treatment for restless leg syndrome (RLS); however, various adverse effects are associated with long-term therapy, and the benefits disappear upon discontinuation. To overcome these limitations, interest in traditional East Asian medicine has increased.

**Patient concerns:**

A 72-year-old Asian woman originally admitted for an intracerebral hemorrhage presented with complaints of an unpleasant sensation throughout the body that appeared at night.

**Diagnoses:**

The patient was diagnosed with chronic persistent RLS based on the 2012 Revised International Restless Leg Syndrome Study Group Diagnostic Criteria.

**Interventions:**

The patient was treated with extracts of the traditional herbal medicines Dangguijakyak-san (DS) and Shihogyeji-tang (ST). After 47 days of therapy, all herbal medicines were discontinued, and symptoms had not returned by the last follow-up 244 days after the initial treatment.

**Outcomes:**

One week after initiating herbal treatment with DS and ST, the RLS symptoms began to improve, and the total hours of sleep had increased from 2 to 9 hours by day 21, with a Korean version of the international restless legs scale score of 11 points. On day 36, ST was discontinued, given the continued improvement of symptoms. On day 47, symptoms had disappeared (Korean version of the international restless legs scale score: 0), and sleep disturbances caused by RLS had completely resolved. After day 47, DS was also discontinued. There were no adverse effects associated with the administration of DS and ST, and the symptoms had not recurred by the last follow-up on day 244.

**Lessons:**

In this case, RLS related symptoms, which had been present for approximately 60 years, were improved using only the traditional herbal medicines DS and ST (without dopamine replacement), and no symptoms recurred for 244 days. This case suggests that if replacement therapy is difficult or not desired, herbal medicinal therapies may be an effective alternative. This also suggests that the effect of herbal medicine on RLS might be semi-permanent. Further investigations, including clinical trials, are needed to confirm these effects.

## 1 Introduction

Restless leg syndrome (RLS) is a neurological disorder involving an uncomfortable sensation in the legs that occurs before going to sleep, which causes sleep disturbance.^[1,2]^ The symptoms of RLS do not appear during the day, but show circadian variability and appear in the evening, resulting in sleep disturbances that reduce the patient's quality of life.^[1,2]^ In Europe and the United States, the prevalence is 7.2% to 11.5%,^[3–8]^ with 7.5% meeting the diagnostic criteria and 1.48% reported to have moderate to severe symptoms.^[9]^ Although it is a relatively common disease, awareness remains low. It has been reported that only about 16% of all patients with RLS receive appropriate treatment.^[9]^

Iron and dopamine are known to play major roles in the pathophysiology of RLS, but the exact mechanism is unknown.^[10]^ Dopamine replacement therapies, such as levodopa and dopamine agonists, are mainly used for symptom control; however, when they are taken for a long period of time (several months or more), augmentation may occur, which worsens the symptoms.^[11]^ Therefore, there are limitations to the long-term treatment of RLS.

To overcome these limitations, interest in traditional East Asian medicinal treatment, such as acupuncture and herbal medicine prescriptions, has been increasing in East Asia.^[12,13]^ Recently, there have been reports that traditional East Asian medicinal treatments, such as acupuncture^[14]^ and herbal medicine,^[15,16]^ are effective adjunctive therapies for RLS.

In this case report, we present a case of RLS that had lasted for approximately 60 years and was treated successfully using herbal prescriptions. After the administration of herbal medicine prescriptions, sensory symptoms in the bilateral calves were dramatically controlled, without recurrence for 6 months. As a result, sleep disturbances, which had been present for approximately 60 years, dramatically improved.

## 2 Case report

A 72-year-old Asian woman had been admitted to our department 28 days previously for treatment of an intracerebral hemorrhage (ICH). During her stay, the patient complained of an unpleasant sensation in her whole body that had been present at night since admission. When the patient was at rest, discomfort and irritation occurred in both calves. All symptoms were resolved by moving the legs. The symptoms mainly occurred in the evening, around dinner time (about 6 pm) and reached a peak during sleep. As a result, the patient could not easily fall asleep, and often woke up during the night. In addition, the patient had to move her legs 7 to 8 times a night because of the discomfort in both calves during sleep. A detailed interview revealed that unpleasant sensations when resting or sleeping had occurred since the patient was an adolescent. However, the patient reported that both her mother and sister had the same symptoms and she had concluded it was a familial characteristic. Therefore, the discomfort in the lower extremities had never been assessed, and had she had never received any treatments or medications to manage it. The patient stated that she had to move throughout the night, and that when someone else was nearby, she refrained from moving, making the discomfort worse. Therefore, she slept alone, even after she was married.

**Figure 1:**
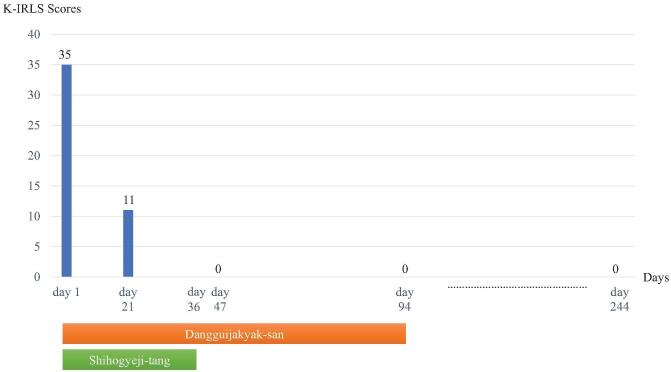
Changes in the Korean version of the international restless legs scale (K-IRLS) score over time.

**Figure 2:**
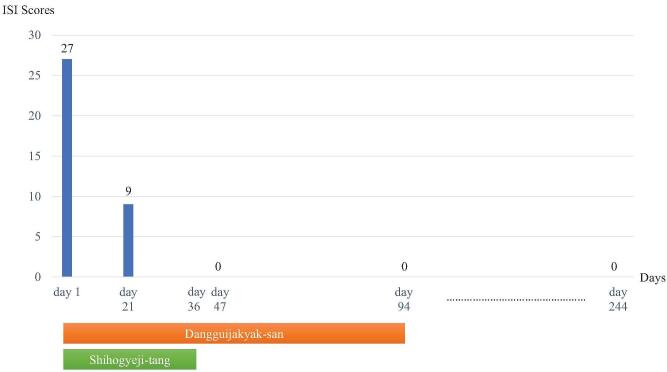
Changes in the insomnia severity index (ISI) score over time.

We diagnosed the patient with chronic persistent RLS based on the 2012 Revised International Restless Legs Syndrome Study Group Diagnostic Criteria.^[17]^ Blood tests revealed no iron deficiency or renal function abnormality. HbA1c was also confirmed to be within the normal range, and diabetes mellitus was excluded. In addition, secondary RLS was excluded because there was no history of peripheral neuropathy, Parkinson disease, or the use of medications known to block the action of dopamine. At the first visit, the Korean version of the international restless legs scale (K-IRLS) was used to evaluate the degree of subjective symptoms,^[18]^ which were found to be the most severe, with a total score of 35 points. To evaluate the effects of RLS on sleep disturbance, the insomnia severity index (ISI) was used,^[19]^ which showed a severe state of sleep disturbance with a total score of 27 points (Figs. 1 and 2).

The primary purpose of admission was to treat left-sided weakness caused by the ICH. However, given the persistence of RLS-related symptoms and subsequent decrease in daytime activity, the patient had difficulty participating in a scheduled rehabilitation treatment program. Therefore, to treat the left-sided weakness, the patient began a rehabilitation program and acupuncture therapy (twice a day), while herbal medicinal treatment was planned to control the symptoms of RLS. The patient had a low weight (159 cm/49 kg), an anorexic tendency, and a pale complexion, with flushing occurring in the afternoon. She had a floating and rapid pulse and a pale red tongue. We therefore determined the patient to have Xue deficiency and Qi stagnation using the identification pattern of traditional East Asian medicine, considering the symptoms of RLS and the aforementioned findings. Among the herbal complexes containing Paeoniae Radix, Dangguijakyak-san (DS, 6 g/d, Kracie; Tokishakuyakusan in Japanese, Danggui Shaoyao San in Chinese) and Shihogyeji-tang (ST, 6 g/d, Kracie; Saikokeishito in Japanese, Chaihu Guizhi Tang in Chinese), which can be used for Xue deficiency and Qi stagnation, were administered to control the symptoms of RLS (Table 1). During the treatment period, other medications the patient was taking were continued, though some were adjusted as needed.

**Table 1:** Composition of Dangguijakyak-san and Shihogyeji-tang.

Latin name	Composition (g/pack)
Dangguijakyak-san	
Paeoniae Radix	3.0
Atractylodis Rhizoma	2.0
Poria Sclerotium	2.0
Alismatis Rhizoma	2.0
Angelicae Gigantis Radix	1.5
Cnidii Rhizoma	1.5
Shihogyeji-tang	
Bupleuri Radix	2.5
Pinelliae Tuber	2.0
*Cinnamomi Cortex Spissus*	1.25
Paeoniae Radix	1.0
Scutellariae Radix	1.0
Ginseng Radix	1.0
Zizyphi Fructus	1.0
Glycyrrhizae Radix et Rhizoma	0.75
Zingiberis Rhizoma Recens	0.25

During treatment, changes in the patient's symptoms and sleep status were assessed daily. The K-IRLS and ISI, which were used to evaluate the severity of RLS on day 1, were used twice more to quantitatively evaluate the changes in symptoms over time (days 21 and 47).

On day 1, the unpleasant sensations were present on the bilateral calves, thighs, ankles, and wrists and the total duration of sleep was 2 hours. One week after the start of DS and ST, the symptoms of RLS began to improve. After 3 weeks (day 21), a significant improvement in symptoms was observed, with the duration of sleep lasting 9 hours. In addition, on day 21, the patient reported that she awakened 2 to 4 times during the night, but that after a brief massage of her legs, she fell back to sleep immediately. On day 36, ST was discontinued given the continued improvement of symptoms. On day 47, symptoms had almost completely disappeared, with no awakening at night, and a duration of sleep averaging 10 hours or more. After day 47 (day 94), DS was also discontinued since there were no further symptoms. According to the K-IRLS score, the symptoms were rated as 11 points on day 21 (moderate) and 0 on day 47 (no symptoms). The ISI results also improved, with 9 points on day 21 and 0 points on day 47. Additionally, the sleep disturbances caused by RLS had also completely resolved. There were no adverse effects associated with DS or ST during the treatment period (Figs. 1 and 2).

The patient's ICH treatment continued until day 86, at which time the patient was discharged. There was no recurrence of RLS by discharge, and approximately 6 months after discharge (day 244), telephone counseling revealed symptoms had still not recurred (Figs. 1 and 2).

On the last day of treatment, the patient signed informed consent for publication of the case report, which was approved by the Institutional Review Board of Kyung Hee University Korean Medicine Hospital (KOMCIRB 2020-05-001-001).

### 2.1 Patient perspective

The following is a qualitative description of the experience of the patient and her caregivers during the course of treatment.

Day 1, patient: “If someone is lying next to me, I cannot sleep. I have to move from time to time, but it is hard because I cannot. So, I have always slept alone.” “It has been harder since the stroke. I guess I cannot move freely because of left-sided weakness.” Her husband: “My wife has too much sensitivity. In the evening, the sensitivity worsens, and she hates to have someone next to her. But all of her family members are like that.”

On day 7, patient: “For the first time, I felt the discomfort at night diminishing.”

On day 21, patient: “Previously, there was discomfort in the calf, thigh, ankle, and wrist, but now only in the calf and thigh.”

On day 47, patient: “I can sleep well without waking up these days.”

On day 86, patient: “I think I can sleep no matter who is next to me.” Her husband: “I’ve always thought of my wife as an unusually sensitive person, but I never thought it was a disease.”

## 3 Discussion

RLS is accompanied by a variety of psychosomatic symptoms, including unpleasant sensations in the legs, sleep disturbances, anxiety, depression, and somatization tendencies.^[20]^ Therefore, patients with RLS have been shown to have a marked deterioration in quality of life compared with healthy populations,^[20]^ but are usually not actively receiving treatment because they do not recognize the symptoms as a disease.^[9]^ In addition, since the mechanism of the disease is not well-known, treatment is aimed at relieving symptoms. Dopamine agonists, levodopa, and anticonvulsants are mainly used to control symptoms; however, they can cause adverse effects such as augmentation, antagonism, dizziness, digestive problems, and dependence.^[21]^ In the present case, the patient had had symptoms of RLS for about 60 years (since she was an adolescent), but neither she nor her family recognized it as a disease and none had ever sought treatment. During the observation time before treatment, the symptoms worsened as left-sided weakness occurred after the ICH. Furthermore, persistent sleep disturbances caused a decrease in daytime activity, which affected stroke rehabilitation. In this situation, both the patient and the family were concerned about the adverse effects of Western medicines. Therefore, DS and ST were considered as an option to control the symptoms of RLS.

After DS and ST were administered for a total of 47 days, the abnormal sensations that had occurred during rest or at night disappeared, and the sleep disturbances had also improved. Thereafter, the administration of both DS and ST had been discontinued, and there was no recurrence of symptoms up to the last day of follow-up, 244 days after the treatment had been initiated. Additionally, no significant adverse events were observed during the treatment period.

DS and ST have a number of pharmacological effects. A previous study suggested that DS has a synergistic effect on the synthesis of acetylcholine and norepinephrine in the cerebral cortex and hippocampus.^[22]^ This pharmacological action is assumed to have a positive effect on climacteric disorder models.^[22]^ ST is a combination of Soshihotang and Gyejitang, and Soshihotang is known to exhibit antidepressant effects by increasing the levels of monoamine neurotransmitters.^[23]^ Therefore, we predicted that combining DS and ST would have a positive effect on RLS, as revealed by psychosomatic symptoms. In particular, we focused on Paeoniae Radix, which is a common component of DS and ST. A systematic review and meta-analysis reported that herbal medicine prescriptions containing Paeoniae Radix were helpful for alleviating symptoms of RLS.^[13]^ Paeoniflorin, the major component of Paeoniae Radix, is known to act as an activator of the adenosine A1 receptor (A1R), which plays a role in stabilizing brain metabolism by lowering synaptic secretions.^[24,25]^ Iron deficiency has often been found in patients with RLS, and an experimental study suggested that an iron-deficient diet lowers the activity of the A1R and dopamine D2 receptors.^[26]^ Based on these findings, we expected that the paeoniflorin in Paeoniae Radix could enhance dopamine activity through the activation of the A1R and recover the function of the dopaminergic A11 system to alleviate the symptoms of RLS.

These pharmacologic effects of DS and ST seem to be involved in the suppression of abnormal sensations related to RLS. Notably, in contrast to conventional pharmacological treatment, abnormal sensations did not recur for months following the discontinuation of DS and ST. Pharmacological treatment with dopamine agonists is known to cause dopamine agonist withdrawal syndrome^[27]^ and RLS symptoms reappear as soon as treatment is discontinued.^[28]^ This also suggests that the components of DS and ST, especially Paeoniae Radix, have a semi-permanent effect on the improvement of A1R function in RLS patients. However, additional clinical and experimental studies are required to further evaluate this potential effect.

## Author contributions

**Conceptualization:** Yuna Seo, Seungwon Kwon.

**Data curation:** Yuna Seo, Seungwon Kwon.

**Funding acquisition:** Seungwon Kwon.

**Resources:** Yuna Seo, Seungwon Kwon.

**Supervision:** Seungwon Kwon.

**Validation:** Yuna Seo, Seungwon Kwon.

**Visualization:** Yuna Seo, Seungwon Kwon.

**Writing – original draft:** Yuna Seo, Chul Jin, Seungwon Kwon.

**Writing – review & editing:** Chul Jin, Bo-Hyoung Jang, Jin Pyeong Jeon, Ye-Seul Lee, Seung-Bo Yang, Woo-Sang Jung, Sang-Kwan Moon, Ki-Ho Cho, Seungwon Kwon.
